# References for Medical Posts–How Best to Utilise Them

**Published:** 1991-03

**Authors:** H. K. Bourns

**Affiliations:** Emeritus Consultant General Surgery - Bristol District Health Authority (Teaching)


### References for Medical Posts — How Best to Utilise Them

**H.K. Bourns, MB, BCh, FRCS.**
Emeritus Consultant General Surgery –
Bristol District Health Authority (Teaching)

I am sure that members of the medical profession are well aware at an early stage in their career of the necessity to develop a skill in the methods of conducting interviews – an article in the BMJ was devoted to a consideration of the rules for such occasions,[1] and much has been written since. The panel which interviews candidates for jobs has a relatively easy task which does not usually generate much toil and emotion apart from the study of the candidates' curriculum vitae. This is often a task to which it is difficult to devote adequate time amongst all the other things that have to be done. Thus it is essential that applicants should not only do the best for themselves but also for the members of the interviewing panel.

It is the general practice to ask candidates for medical jobs of all categories, apart from some pre-registration posts, to provide the names and addresses of two or more persons who will be prepared to give an opinion in writing on their character, ability and suitability for the post for which they have applied. Since medical positions are in such short supply relative to the number of candidates suitable qualified, the selection of a referee and his response is more important than ever. Therefore, the candidate and the referee must have a very close liaison, and the value of a reference must be clearly understood by both parties.

Application for a post should be related to the needs and training of a particular doctor. He or she should, in an ideal world, discuss the next move in the chosen career with the referee, and thus a reference will not only describe the candidate as known to the referee, but will allow the description of the job in question to be taken into detailed consideration at that time in relation to the candidate's experience and needs.

At the outset one must state that no referee should give a candidate an adverse reference without informing him or her that he feels unable to help in the application. To allow a person to go forward for a job unaware that a reference is not in his favour is most unfair. A referee must always respond to a request if a candidate insists, but to give a poor opinion without the candidate's knowledge is likely to damage a person's career and may prevent further training, particularly if the reference is used in a number of applications. Candidates must realise that the change from open testimonials to references known only to the Appointment Committee was largely designed in order that honest and straightforward opinions could be expressed. Therefore, all applicants for posts must see to it that they do have the full support of those whom they ask to speak for them.

There are two points to be made about this position. Firstly, candidates must always contact the referee either personally or by letter when they want a reference. They cannot expect anything very helpful or detailed if the first knowledge that a referee receives in relation to a post for which he is expected to give a useful reference is a request from the Authority which has advertised the post. This fact alone may so irritate a potential referee that he may write a reserved opinion because he feels that the candidate has been both discourteous and high handed. Referees should be given the opportunity to discuss the reason why an applicant is seeking a particular job and all referees must be prepared to give some guidance to the doctor about the value of a job in relation to a career. All applicants must keep in touch with those to whom they are looking for support, and all referees must give of their experience and time to advise them as to the various steps that might be taken at that particular point. For instance, a particular post might be more useful after a person has completed an examination for a higher degree rather than before. Many jobs are so busy that study is impossible and study leave is known to be unlikely so that the order in which a person obtains the posts which will further his training is always worth consideration.

Secondly, a candidate should always refer to one of the consultants with whom he has been working recently. To continue to use a person with whom one has not worked for some years may be justified if that referee can advise on experience in a particular speciality, but unless there is some overriding position, Committees always notice and are possibly disturbed when a candidate presents himself with references which come from consultants with whom he has not worked recently. When there are so many good candidates for posts then the Appointing Committee looks at absolutely every aspect of a candidate's past and present as outlined in his or her curriculum vitae, and usually discusses the omission of references from present or immediate past consultants.

It is worth pointing out that most candidates only obtain an interview because they have not only carried out certain posts but because they have presented a clear and well prepared account of their experiences, prizes and qualifications up to that point. If there are any gaps or other unusual features about their previous activities these must not be hidden but must be presented in the best possible light. Candidates must consult their referees about their curriculum vitae so that they may appear to make the most of their available experience. There must be no suggestion of any concealment of any unusual features. To attempt to mislead a Committee can only be detrimental. A full exposure of all points is the right policy. The candidate will, of course, be in the best possible position to explain what has happened in the past if it is plainly stated and is equally satisfactorily explained. The committee will immediately be on the candidate's side if they, together with the help of the references, realise the facts and the reasons for them. A candidate must consult with the referee not only about the job for which he is applying but must also seek advice on the presentation of his experience up to that point. To obtain an interview for a particular post depends in the first instance on a clear and succinct presentation. It must not be too long because one has to remember that a clear account bringing out all the essential facts without unnecessary complications is the one that a member of an Appointment Committee is likely to consider most favourably. If one has the experience that is likely to bring short listing it means that the course of a career up to that point is reasonable and the candidate must see to it that his referees are in touch with both his work and aspirations and the benefit likely to follow the anticipated next stop.

It can happen that a candidate is frequently short listed for a number of similar posts but is always unsuccessful in the appointment. The likely causes are either that his interview technique is poor or one or both of his or her referees do not give a favourable reference.

If a person is always short listed but never successful he should seek the advice of one or both referees. It might be that they are not very happy with the candidate's demeanour or attitude and might even have heard why he or she was not appointed. They might have known a member of the Appointing Committee and whilst the proceedings are always confidential some important point might be passed on to help someone who was close to success. A referee might even arrange for a trial interview for a candidate both to assess his attitude and to instruct in technique. So often a candidate is worried about the questions asked at an interview and spends time and thought wondering where the catch is when in fact it is a straight but probing question to which he should give a factual and honest answer. An opinion will reveal the candidate's potential. Any idea which is put forward must then be discussed as fully and completely as the Committee seems to desire.

There is no point in giving evasive answers because such an attitude will be exposed and will only have a detrimental effect. A person will usually know when an interview has gone badly and must consider most urgently the reason.

If one is repeatedly unsuccessful then it may be that the referee should be reconsidered. If it is known that one in particular is rather reserved or critical, then it might be wise to employ another. Referees are known to Committees and so this sort of situation is likely to be well understood, but nevertheless, repeated lack of success which is not due to poor interview technique is likely to be related to the selection of referees for a particular job. It can be seen how important it is to have both respect for and regular contact with one's referees in order to increase the chance of success, and any suggestion of reserve by a referee for a particular post should be noted and the reasons should be discussed and sorted out beforehand if this is possible.

The present system of making medical appointments is about as satisfactory as any that could be devised, but it would appear that some candidates for all sorts of posts are neither fair to their referees nor fair to themselves. Career guidance is more essential now than ever it was, and candidates should seek advice when they wish to apply for a post. They do not necessarily have to take advice but they cannot expect a referee who has advised them not to seek a particular post to give an enthusiastic opinion if he or she does not think that the job is either satisfactory in itself or the right appointment at that particular point.

In every major centre where there are a number of trainees there are also tutors appointed by the Royal Colleges. When a junior doctor works in a centre he or she should make himself known to the local College Representative. The council of any particular Royal College may change regulations at any time. These changes are always circulated, but a publication may easily escape notice so that contact with the local tutor is both necessary and desirable.

There are many more locum jobs advertised now than ever before, and trainees should seek advice before they embark on either one or a series of locum appointments. To take this course usually interferes with training, and anyone who has a number of locum posts in their curriculum vitae should always be ready to give a reason why that course was taken.

It is essential to have an up-to-date curriculum vitae prepared for the job in question. All information must be accurate, and any achievement outside the normal run of career jobs should be included. It will always interest the Committee members and give an indication of a particular interest or unusually successful aspect likely to relate either to a candidate's personality or initiative. A copy of a duplicated application which may indicate many applications suggests that a candidate is not having much success or indeed is not expecting to have much success in a particular post. One must always give the impression that this is the job that one wants above all else.

If a candidate who is short listed cannot attend an interview on a particular date or time he or she must always give as much notice as possible, and in many cases an alternative arrangement may be possible. If someone does not let the Health Authority know that he cannot attend an interview and just does not turn up, this will be noted and stand against that person on future occasions. Always inform the Regional or District Health Authority as soon as it is clear that a particular interview date cannot be kept.

Selection of a referee is usually easy. He or she is one for whom one has worked, and whose speciality is related to the job in question. If, however, it transpires that a referee is going to be a member of the Appointment Committee for the job when he will speak favourably for the candidate it is usually wise to pick another person who will not be present to give a reference so that there will be the required number of written views desired by the Committee. Thus, a candidate will not suffer in comparison with the others as far as outside reports are concerned. There is such intense competition for most jobs that attention to detail is essential, and consultation with the College Tutor, referees and present consultant chiefs should not be neglected. Success is the result which is always desired, but slack actions and evidence of a careless approach may even prevent an applicant from being short listed much less being appointed to the post for which he or she has applied. Canvassing is not permitted in the United Kingdom, but a visit to a hospital and discussion with members of staff both junior and senior is in order. No-one should wait until he reaches an Appointment Committee to find out about a particular job. Failure to ascertain the implications of a post before attending does suggest that a candidate is not very interested in it because it is evident to the Committee that essential knowledge has not been sought before reaching the interview. It is nearly always possible to have time off to visit a hospital to seek information about a post. If a candidate cannot do so he ought to telephone in advance or give the reason at the interview either to the secretary prior to the meeting or at the Committee when the opportunity present.s The acquisition of essential information about any job will not be considered to be unfair canvassing.

A lecture[2] given at a National CMF Conference on this subject by a Professor of Gastoenterology in the University of London and Consultant Physician to the London Hospital emphasises many of the points that have already been discussed. In the conclusion of his lecture he states that the candidate must take trouble over the application. If careless, untidy or incomplete they will give a bad impression and a good candidate may not be shortlisted because of this alone. He also points out that the candidate must "show that he wants the job". Take trouble in preparation for an interview and put one's whole personality forward at the time. In this way the members of the panel will be able to judge on both technique and quality. Never accept the fact that a job is "fixed". Take the opportunity offered by an interviewing panel to prove oneself to be the best in the field.

### REFERENCES

1. GEORGE DICK, Conduct an Interview, *Br. Med. Journal*, 1979 **1**, 42–43.
2. JOHN LENNARD-JONES. In The Service of Medicine – 1984. Vol. 30: **4** No. 120, 13–17.

